# Spirituality in palliative care: From assessment to existential presence

**DOI:** 10.1017/S1478951526103162

**Published:** 2026-09-03

**Authors:** Evandro Leite-Bitencourt, João Carlos Geber-Junior, Julia de Camargo, Alan Roger dos Santos-Silva

**Affiliations:** 1 Hospital Sirio-Libanes, Faculdade Sírio-Libanês, São Paulo, SP, Brazil; 2 Palliative Care Team, Hospital Samaritano Higienópolis, São Paulo, SP, Brazil; 3 Faculty of Medicine, University of São Paulo, São Paulo, SP, Brazil; 4 Oral Diagnosis Department, Piracicaba Dental School, University of Campinas, São Paulo State, Brazil

Spirituality is formally recognized as a core domain of palliative care. Yet recognition alone has not ensured integration. In clinical practice, spirituality often remains peripheral – confined to screening instruments or delegated exclusively to chaplaincy services. This essay proposes that spirituality should not be understood merely as a dimension to assess, but as the existential and relational field in which suffering, meaning, identity, and dignity converge in serious illness.

The World Health Organization defines palliative care as addressing physical, psychosocial, and spiritual suffering as inseparable dimensions of human experience (World Health Organization 2018). Despite this formal inclusion, spiritual care is frequently approached instrumentally. Structured tools such as the FICA Spiritual History Tool (Puchalski et al. 2009) offer important entry points, yet tools alone cannot sustain the depth of engagement required when patients confront finitude.

Spiritual distress rarely presents as a discrete symptom. It emerges instead in questions without diagnostic endpoints: Why is this happening to me? Who am I if I can no longer fulfill my roles? What remains meaningful now? These are not biomedical queries but existential appeals. When such appeals are met only with clinical efficiency, the deepest layer of suffering risks remaining unaddressed.

## A clinical encounter

A patient with metastatic gastrointestinal cancer presented with reasonably controlled physical symptoms. Pain was manageable; dyspnea was mild. Laboratory markers were stable. During a routine consultation, she asked quietly, “If I am no longer able to care for my grandchildren, who am I?”

No imaging study could clarify that question. No laboratory parameter could interpret it. What followed was neither theological instruction nor psychiatric categorization, but a deliberate pause. The conversation turned toward identity beyond productivity, dignity beyond function, and love beyond physical capacity. Her anxiety softened – not because her prognosis changed, but because her suffering had been acknowledged as meaningful.

Such encounters resonate with prior reflections in this journal describing how palliative care unfolds within suspended time, where uncertainty becomes the space of relational presence (Geber-Junior and Forte 2025). Spirituality frequently emerges within this temporal suspension, not as doctrine but as narrative reconstruction.

## Spiritual well-being as clinically consequential

The work of Breitbart et al. (2010) has been instrumental in establishing spirituality as clinically consequential. In a landmark study, McClain et al. (2003) demonstrated that spiritual well-being was among the strongest predictors of lower hopelessness and reduced desire for hastened death in terminally ill cancer patients. This finding repositioned spirituality from abstract humanism to a measurable clinical correlate of existential outcomes.

Meaning-centered psychotherapy, subsequently developed by Breitbart et al. (2010), demonstrated that meaning can be actively cultivated even in advanced illness, leading to improvements in spiritual well-being and reductions in existential distress. These interventions do not impose belief systems; rather, they facilitate narrative coherence when illness disrupts identity.

More recent scholarship emphasizes that effective palliative care communication requires attention not only to informational needs but also to patients’ values, emotions, relationships, and existential concern (Engel et al. 2023). Within this broader communicative context, spiritual suffering may influence how patients and families interpret illness, express treatment preferences, and engage in end-of-life deliberation. To omit spirituality from serious illness care is not neutrality; it is the neglect of a potentially modifiable dimension of suffering.

Complementing this body of evidence, a recent synthesis of 3 decades of research led by Ferrell examined spirituality across the cancer trajectory and consistently identified it as a central dimension of patient experience (Ferrell et al. 2024). Spirituality emerged not merely as religious belief but as an expansive construct encompassing meaning, purpose, hope, faith, and relational connection. These dimensions were significantly associated with quality of life and psychological well-being, while spiritual distress correlated with heightened emotional burden. Importantly, the synthesis highlighted persistent gaps in clinicians’ preparation to address spiritual concerns in routine practice – underscoring the need to move from nominal recognition to relational integration.

## Distinguishing pathology from existential grief

A recurrent risk in contemporary medicine is the medicalization of existential suffering. Not all despair represents major depressive disorder. Not all silence indicates pathology. As previously reflected in accounts of diagnostic waiting and anticipatory grief (Curatolo and Geber-Junior 2025), suffering may represent mourning unfolding in real time rather than psychiatric dysfunction.

Distinguishing clinical depression from existential grief requires what may be termed existential literacy: the clinician’s capacity to recognize meaning-related suffering, tolerate ambiguity, and remain present without prematurely pathologizing vulnerability.

Existential literacy does not replace psychiatric rigor; it refines it. It enables differentiation between demoralization and depression, spiritual distress and cognitive disorder, grief and psychopathology – distinctions with significant therapeutic implications.

Existential literacy, however, must be cultivated rather than presumed. While relational presence cannot be reduced to a checklist, structured approaches to spiritual history-taking offer ethically grounded entry points into conversations that might otherwise remain unaddressed. Taking a spiritual history enables clinicians to understand how patients construct meaning in the face of illness, sustain personal dignity, and mobilize inner resources throughout the disease trajectory.

Several structured models have been developed to guide clinicians in this process, including FICA (Puchalski 2014), HOPE (Anandarajah and Hight 2001), SPIRIT (Maugans 1996), FAITH (Neely and Minford 2009), and frameworks proposed by professional bodies such as the Royal College of Psychiatrists (Culliford and Powell 2006). These models support exploration of belief systems, sources of meaning and strength, religious or spiritual expressions, and the ways in which these dimensions shape coping strategies and healthcare decisions.

Tools do not replace presence; they prepare the ground for it.

### Spirituality, vulnerability, and relational ethics

Serious illness exposes the limits of autonomy conceived solely as decisional capacity. As vulnerability intensifies, patients often seek not procedural clarity but narrative coherence. Spirituality, in this context, becomes less about belief systems and more about recognition – being seen as a person whose suffering carries meaning beyond physiology.

Relational scholarship in palliative care increasingly recognizes that the heart of the field lies not solely in intervention but in sustained engagement (Engel et al. 2023). Prior reflections describe how care unfolds within shared uncertainty rather than predictive certainty (Geber-Junior and Forte 2025). Spirituality operates precisely within this relational ethic.

When families speak of miracles, divine timing, or transcendent hope, they may not be rejecting medical reality; they may be articulating resilience. Responding with corrective data alone risks symbolic rupture. Engagement, rather than confrontation, preserves therapeutic alliance.

## From domain to atmosphere

Spirituality in palliative care should therefore be reframed – from a domain of assessment to the atmosphere in which whole-person care unfolds. It is not an optional sensitivity appended to biomedical competence; it is the relational medium through which suffering becomes intelligible.

Integrating spirituality does not dilute scientific medicine. On the contrary, it refines it. When clinicians remain present amid uncertainty, validate existential questions, and facilitate meaning-making without correcting hope into submission, they address dimensions of suffering empirically linked to hopelessness, desire for hastened death, and quality of life.

Cure may not be possible. Control may be partial. Meaning, however, remains dynamic.

Spiritual care is not about providing definitive answers. It is about sustaining presence where answers fail – and recognizing that, in serious illness, presence itself may be the most rigorous form of care.
